# The Best Women in the Kingdom

**Published:** 1920-02-28

**Authors:** 


### SHOULD MATRONS BE ELECTED?

### The Best Women in the Kingdom.

THE College elections are approaching, and, as *via* the College lies the way of a nurse's salvation, the incident, whenever it occurs, is of the utmost importance.

A great many people think that the College Executive is overweighted with hospital matrons, and they propose that nurses who are not matrons should be elected in their stead. I have always been an advocate of the rank and file of the profession, but I confess that I am unable to follow the logic of this argument. I think that the people who make such a recommendation are conscious that there is a defect, but it is extremely unlikely that such procedure as substituting non-matrons for matrons would effect a cure. Anyhow, I propose to set out my views on the subject, and I hope others will follow my example.

### WHAT IS WRONG?

To begin with, what, if anything, is wrong? The College has accomplished a vast deal since its establishment, and it may be unkind to suggest that everything is not as it ought to be. My experience is that criticism is helpful and wholesome, and I do not think that the College Executive need wince if it is pointed out that it is not an absolutely perfect corporation. My criticism is that the members of the College Executive are not in close touch with their constituents. The fact is that the requirements of nurses are very numerous. They want scores of things reformed and improved, and, although the College is always moving, there is a general consensus of opinion that the gait is too leisurely, and that, moreover, the ladies who form the Executive are not, or do not appear to be, aware that the rank and file are chafing at the slow movement. Am I accurate when I say that the Executive, collectively or individually, have never gone to the nurses as a body, as a Member of Parliament goes to his electorate and puts to them the question, "What do you want me to do for you"? The College of Nursing, whatever else it may be, is, without doubt, a nurses' parliament, and whenever a man or a woman becomes a Parliamentary candidate a full confession of faith is absolutely essential at the hustings. I venture to prophesy that in the future none will be more exacting in this respect than the woman elector. She will have crystallised in her mind exactly what she wants to accomplish, and she will demand that her representative speaks with no uncertain voice. She will make herself felt in Parliament. It was the women's vote that made America a "dry" country, and, if she does not make England "dry," she will certainly curtail the vintage.

### ROOM FOR CRITICISM.

I venture to ask the College Executive if they are conscious of having ascertained from British nurses what reforms they are desirous of effecting. Are these catalogued in order of importance, and are strenuous efforts being made to make them accomplished facts? And, if the Executive have in truth used the maximum of effort, have they taken the nurses into their confidence and demonstrated that it has not been possible to do more? My own opinion is that there is room for criticism, and when people talk about evicting the matron, and putting a subordinate nurse in her place, I believe that they have a vague and indefinite feeling that the matron is either too timid or too conservative.

Is it reasonable, may I inquire, to suppose that a nurse who is not a matron would make a better executive officer? I do not think so. I believe that the matrons as a body are the cream of the profession. They have highly-responsible duties to perform, which have nothing to do with nursing. It is possible to become a first-class nurse with one talent. The successful matron of a cottage hospital must have, at least, two talents. The matron of a Metropolitan hospital must have, at least, ten talents. Is it not so?

Another consideration. It is, no doubt, a great honour to be elected a member of the College Executive. But it is a thankless job—for proof of which please note my criticisms. I can visualise many members of the College Executive exclaiming at my audacity in suggesting lack of energy and lack of other things. If they cherish resentment, I freely admit that they are fully justified. Why should these hard-worked and worthy women raise a finger? From this view-point I think it must be universally acknowledged that they have worked wonders. Why should Sir Arthur Stanley devote his time, his talents, and his energy to the betterment of British nurses? If we are critical, let us be just, and, also, let us be grateful. These people have no axe to grind. They are inspired by the highest motives.

### THE BEST WOMEN NEEDED.

I fully recognise all this. And I also recognise that the nurse has many and grave grievances which loudly call for redress. I am as convinced as I can be of anything that she can have them redressed, and speedily, always provided that the subject is properly placed before the British public. I give way to none in my appreciation of the British soldier. But I hold the view that the British nurse is at least as important to the nation as the soldier. It is only when the drums begin to roll that we think of "the thin red line of 'eroes." If the nation is to be efficient, and is to maintain its place in the sun, we must have more nurses, better nurses, happy and contented nurses, in the piping times of peace.

I am not talking sentiment—I am talking business; I am talking economics. I have always held the opinion, and I hold it still, that it was a colossal mistake on the part of the College of Nursing to charge only a fee of a guinea for life membership. A guinea per annum might be too much, but it would be much more sensible than a guinea for evermore.

I hope I may not hurt anybody's feelings, but my ideal Executive Committee is a Committee of Matrons and public women, paid, and generously paid, for their services. A College of Nursing is a proud possession. But is there any other unpaid College in England? I know of none. It is the obvious duty of the nurse to rally round her College and to support it. The best women in the Kingdom should be on the Executive, and even if money is no object, the nurse should refuse to elect candidates who decline to receive remuneration for their services.

To depose a matron because she is a matron is tosh.

IERNE.

